# A Manual of Surgical Treatment

**Published:** 1901-11

**Authors:** 


### BOOK REVIEWS.

A Manual of Surgical Treatment. By W. Watson Cheyne, M. B., F.R. C.S., F.R.S., Professor of Surgery in King's College, London, Surgeon to King's College Hospital, etc.; and F. F. Burghard, M D and M. S. (Lond.), F. R. C. S., Teacher of Practical Surgery in King's College, London, Surgeon to King's College Hospital, etc. In seven imperial 8vo volumes, with illustrations. Volume V., 482 pages with 145 illustrations. Philadelphia and New York: Lea Brothers & Co. 1901. [Price, cloth, $5.00 net.]

Cheyne and Burghard do not show any signs of getting tired as their work proceeds, and the fifth volume comes from the press with all the freshness and enthusiasm which marked the very first of the work. It is rare indeed that one finds any semblance of style in medical publications, or the slightest trace of literary merit; yet in all the work of these two eminent men of England, one detects a distinct flavor of literature in the writing of scientific matters, and in some places it actually bursts forth a delightful bloom of gracefully turned phrases, in which the information imparted is beautifully draped in simple unaffected language, which might well be imitated by some of the more pretentious of the American medical writers.

There are no orthographic gymnastics, no high and lofty tumbling with the English language, but a plain and simple statement of facts and conditions, presented in such a manner as to inspire confidence and respect. There are few if any writers on this side of the great waters who have turned out a work on surgery which can compare with Cheyne and Burghard in this regard. Probably the reason for this is that the greater number of works in this department have been of a composite character—systems in which a dozen or more men have had a hand, and which very naturally under the circumstances were nothing more or less than scientific and literary patch work. Individually, each department of an American work is a jewel; it is so in Park's surgery, and in Agnew's,—works which inspire the confidence which is so necessary and which is so lamentably lacking, so woefully wanting in many American books. Had these two men infused their style into the practice of their co-laborers we would have on our shelves today two works on surgery which would forever be scintillating gems in scientific literature. As it is, they did no such thing and while the works are brilliant individually, it has been left for Cheyne and Burghard to give us a thoroughly comfortable series of volumes on surgery.

They are really "heart-to-heart talks" on surgery and one feels as if he were receiving personal instruction when reading them. The fifth volume, just issued, is divided into two sections. The first deals with the surgical affections of the head and face, and includes the scalp and skull; intracranial injuries and suppuration; hernia cerebri; meningocele, encephalocele and micro- and hydrocephalus; focal epilepsy and brain tumors. Of the affections of the face are wounds, ulcers and new growths; fractures of the nasal bones; affections of the lips; neuralgia; plastic surgery of the face, hare-lip and cleft palate; fractures, tumors and inflammations of the jaws; congenital deformities of the larynx and trachea; cut throat; foreign bodies in the air-passages; operations; cancer.

In the second section devoted to intrinsic diseases of the nose, ear and larynx detail has not been lost sight of, and it has been practically made a specialist's section. For the nose are given the anatomy, examination, deformities, inflammatory affections and sequelæ; chronic infectious diseases and neuroses. The ear and larynx are handled in the same manner of completeness.

In this division, credit for the preparation of which is given to Dr. Lambert Locke, there is not noticeable any difference in the style of presentation. The material may be Locke's but the handiwork of Cheyne and Burghard is plainly in evidence. There are the same gracefully turned sentences, the same confidential tone, the same feeling of personal interest which is so apparent in those portions of the work which are directly from the pens of these English surgeons.

With few exceptions the illustrations are original, and these are the presentation of instruments.

There are two other volumes to be issued before the work is completed and they cannot come too soon.

